# Chlorogenic Acid Targeting of the AKT PH Domain Activates AKT/GSK3β/FOXO1 Signaling and Improves Glucose Metabolism

**DOI:** 10.3390/nu10101366

**Published:** 2018-09-23

**Authors:** Jie Gao, Xin He, Yuejiao Ma, Xuezhi Zhao, Xiaotao Hou, Erwei Hao, Jiagang Deng, Gang Bai

**Affiliations:** 1 State Key Laboratory of Medicinal Chemical Biology, College of Pharmacy and Tianjin Key Laboratory of Molecular Drug Research, Nankai University, Haihe Education Park, 38 Tongyan Road, Tianjin 300353, China; gaojie@nankai.edu.cn (J.G.); xinhe@mail.nankai.edu.cn (X.H.); 2120171270@mail.nankai.edu.cn (Y.M.); zhaoxuezhitj@126.com (X.Z.); 2 Guangxi Key Laboratory of Efficacy Study on Chinese Materia Medica, Guangxi Collaborative Innovation Center for Research on Functional Ingredients of Agricultural Residues, Guangxi University of Chinese Medicine, 13 Wuhe Avenue, Nanning 530200, China; xthou@126.com (X.H.); ewhao@163.com (E.H.); dengjg53@126.com (J.D.)

**Keywords:** chlorogenic acid, glucose metabolism, protein kinase B (AKT), pleckstrin homology (PH) domain, glycogen synthase kinase 3β (GSK3β), forkhead box O1 (FOXO1)

## Abstract

Chlorogenic acid (CGA), a bioactive component in the human diet, is reported to exert beneficial effects on the regulation of glucose metabolism. This study was designed to investigate the specific target of CGA, and explore its underlying mechanisms. Beneficial effects of CGA in glucose metabolism were confirmed in insulin-treated human hepatocarcinoma HepG2 cells. Protein fishing, via CGA-modified functionalized magnetic microspheres, demonstrated the binding of CGA with protein kinase B (AKT). Immunofluorescence using a CGA molecular probe further demonstrated the co-localization of CGA with AKT. A competitive combination test and hampering of AKT membrane translocation showed that CGA might bind to the pleckstrin homology (PH) domain of AKT. The specific binding did not lead to the membrane translocation to phosphatidylinositol (3,4,5)-trisphosphate (PIP_3_), but directly activated the phosphorylation of AKT on Ser-473, induced the phosphorylation of the downstream molecules, glycogen synthase kinase 3β (GSK3β) and forkhead box O1 (FOXO1), and improved glucose metabolism. Collectively, our data demonstrate that CGA exerts regulatory effects on glucose metabolism via direct targeting the PH domain of AKT. This study clarifies the mechanism of the potential benefits of nutrients containing CGA in the complementary therapy of glucose metabolism disorders.

## 1. Introduction

Chlorogenic acid (CGA), a polyphenol with potent antioxidant activity, is an ester of caffeic acid and quinic acid [1]. The consumption of CGA is, or is predicted to be, associated with lower risk of a series of diseases, such as cardiovascular diseases, type 2 diabetes, and Alzheimer’s disease [2,3,4]. CGA possesses many biological activities, including antioxidant, antibacterial, and anti-inflammatory activities. Regular consumption of coffee or tea, two of the most popular daily drinks around the world, was reported to lower the risk of type 2 diabetes mellitus, indicating beneficial effects on glucose and lipid regulation [5,6]. These effects were attributed to the high content of polyphenolic components, most notably, CGA [7,8]. In addition to coffee and tea, CGA is also abundant in other human dietary components, such as fruits and vegetables [8]. Furthermore, CGA is highly absorbed and metabolized in humans, and it retains bioactivity across the intestinal barrier [9,10]. Therefore, dietary nutrients containing CGA might be applied as a promising complementary therapy for glucose and lipid metabolism disorders.

Although the beneficial effects of CGA in the regulation of glucose metabolism in patients was confirmed [11], the underlying mechanisms are still under investigation. For example, in Sprague-Dawley rats fed a high-fat diet, CGA was shown to modulate the expression of certain glucose transporters (sodium/glucose linked transporter 1 (SGLT-1) and glucose transporter 2 (GLUT-2)) and proglucagon, thereby regulating glucose metabolism [12]. Sanchez et al. reported that CGA might serve as a multitarget agent that regulates glucose. In their results, CGA was demonstrated to act as both an insulin secretagogue and a peroxisome proliferator-activated receptor (PPAR)-α/γ dual agonist [13]. It should be noted that the Ser and Thr kinase, protein kinase B (AKT), was recently reported to participate in the regulation of glucose metabolism by many groups [14,15]. However, whether AKT participates in the regulation of glucose metabolism by CGA remains unclear.

As the most important downstream factors of AKT, glycogen synthase kinase 3β (GSK3β) and forkhead box O1 (FOXO1) play important roles in the regulation of glucose metabolism exerted by AKT. GSK3β is the downstream mediator in the insulin receptor substrate (IRS)/phosphatidylinositol 3-kinase (PI3K)/AKT/GSK3β signaling pathway. Through phosphorylation and subsequent inhibition of GSK3β, insulin activates glycogen synthase and lowers blood glucose. Blocking this pathway might result in insulin resistance with impaired glucose metabolism [16,17]. On the contrary, activation of the AKT/GSK3β pathway leads to improvement of glucose metabolism [18]. The AKT/FOXO pathway is another pathway that participates in glucose metabolism regulation. FOXO1 belongs to the FOXO family which consists of several FOXO proteins. They may act as nuclear transcription factors to mediate the inhibitory action of insulin in mammals. During the phosphorylation of FOXOs by AKT, FOXOs interact with the 14-3-3 protein, and are excluded from the nucleus, leading to their eventual ubiquitylation-dependent degradation. When FOXOs are inhibited, glucose production decreases, potentially benefiting diabetes treatment. FOXO1 was reported to play a role in hepatic glucose homeostasis by regulating the gene expression of gluconeogenic and glycolytic enzymes, such as glucose 6-phosphatase (G6P) and phosphoenolpyruvate carboxykinase (PEPCK) [19,20]. Altered expression of the FOXO1 protein was reported to be associated with diseases such as diabetes [21]. Regulation of FOXO1 activity was reported to lead to improvement of glucose metabolism [22]. Furthermore, clinical evidence showing that genetic FOXO1 variants are associated with insulin resistance and type 2 diabetes also supports the contribution of FOXO1 in glucose metabolism in humans [23].

Although CGA is reported to exert beneficial effects on glucose and lipid metabolism through diverse proteins and pathways, the direct targets of CGA remain unexplored. In this study, we tried to identify the direct targets of CGA in its regulation of glucose consumption by integrated chemical biology methods. Then, we elucidated the underlying signaling pathways, with focus on GSK3β and FOXO1.

## 2. Materials and Methods

### 2.1. Materials

Chlorogenic acid (purity >98.5%, determined by high performance liquid chromatography (HPLC) ()) was purchased from Aladdin (Beijing, China). Insulin was purchased from Sigma-Aldrich Co. (St. Louis, MO, USA). Metformin hydrochloride (Met) was sourced from Adams Reagent, Ltd. (Shanghai, China). N_3_-tag (3-azido-7-hydroxy-2H-chromen-2-one) was supplied by WuXiAppTec (Beijing, China). The primary antibodies for glyceraldehyde 3-phosphate dehydrogenase (GAPDH), phosphorylated (p)-AKT, AKT, p-GSK3β, GSK3β, p-FOXO1, and FOXO1, and secondary antibodies were purchased from Cell Signaling Technology (Beverly, MA, USA). Alexa Fluor^®^ 594-conjugated goat anti-rabbit immunoglobulin G (IgG) was purchased from Abcam (Cambridge, UK). Chemiluminescent horseradish peroxidase (HRP) substrates were from Millipore Corporation (Burlington, MA, USA). SC79 and AKT inhibitor VIII were purchased from MCE (Monmouth Junction, NJ 08852, USA). PHT-427 and AT7867 were from Selleck (Shanghai, China). The pcDNA3-AKT-pleckstrin homology (PH)-GFP and pcDNA3-AKT-PH(R25C)-GFP plasmids were purchased from Addgene (Cambridge, MA, USA). All cell culture reagents were purchased from GibcoBRL Life Technologies (Grand Island, NY, USA). All other used chemicals were of analytical grade.

### 2.2. Cell Culture

HepG2 and human embryonic kidney (HEK) 293T cells, purchased from American Type Culture Collection (Rockville, MD, USA), were cultured in Dulbecco’s modified Eagle medium (DMEM) supplemented with 10% fetal bovine serum (FBS), 100 U/mL penicillin, and 100 μg/mL streptomycin. All cells were maintained in a 5% CO_2_ humidified atmosphere at 37 °C.

### 2.3. Glucose Consumption

HepG2 cells were grown in 96-well culture plates at a density of 2 × 10^4^ cells per well. After 24 h, the culture medium was replaced by serum-free medium, with or without insulin, for 24 h. Based on our preliminary experiments, 1 μM was chosen as the concentration of insulin to induce glucose consumption disorder in HepG2 cells. Then, cells in different treatment groups were treated with CGA (100, 10, and 1 μM) or Met (10 μM) for another 12 h. Met was used as a positive control. Following treatment, the supernatants were collected for a glucose concentration assay. Glucose levels were determined using a Glucose Assay Kit (Nanjing Jiancheng Bioengineering Institute, Nanjing, China) based on the glucose oxidase method, according to the manufacturer’s instructions. Glucose consumption was calculated according to a previous article [24]. Glucose consumption was normalized using the 3-(4,5-dimethylthiazol-2-yl)-2,5-diphenyltetrazolium bromide (MTT) assay.

### 2.4. Enzymatic Activity Determination

HepG2 cells were plated in 75-cm^2^ culture flasks at a density of 5 × 10^6^ cells per flask, and grown to 80% confluence. For synchronization, the cells were incubated in a serum-free medium for 24 h, then treated with either CGA (10 and 1 μM) or SC79 (10 μM) for 6 h. The enzymatic activities of AKT and GSK3β in cell lysates were then quantified as described in the manufacturer’s manuals (GENMED, Arlington, MA, USA).

### 2.5. Target Prediction and Molecular Docking

Target prediction and docking of CGA were performed as described in a previously published work from our lab [25]. Briefly, the three-dimensional structure of CGA was input into the PharmMapper database (http://59.78.96.61/phammapper). The related pathways and interactions of proteins were analyzed using the Kyoto Encyclopedia of Genes and Genomes (KEGG) (http://bioinfo.capitalbio.com) and String version 9.1 (http://www.string-db.org/), respectively. The three-dimensional structures of potential protein targets were obtained from the Protein Data Bank (http://www.rcsb.org/pdb). The structures of the molecules and potential targets were constructed using the Sybyl-X 2.0 software package (Tripos International, New York, NY, USA). The calculations of binding energy and molecular docking were performed using AutoDock version 4.2 (Olson Laboratory, LaJolla, CA, USA) and Molecular Operating Environment (MOE 2015.10). The original ligand, inositol (1,3,4,5)-tetrakisphosphate (IP_4_), was docked into the binding pocket of the AKT PH domain as a positive control.

### 2.6. Target Fishing

HepG2 cells were plated in 75-cm^2^ culture dishes, and grown to 80% confluence. Subsequently, the cells were washed three times with precooled phosphate-buffered saline (PBS), and incubated with 0.5 mL of radioimmunoprecipitation assay (RIPA) lysis buffer (China COSCO, Beijing, China) for 30 min at 4 °C. Then, the lysates were collected and centrifuged at 10,000× *g* for 15 min to obtain the supernatants. The protein concentration was quantified using a BCA protein assay kit (Thermo Scientific, Waltham, MA, USA). CGA-modified functionalized magnetic microspheres (MMs) were used to fish the targets of CGA out from the pool of total proteins. An alkynyl-modified CGA probe (alkynyl-CGA), the substrate for obtaining CGA-modified MMs in the click reaction, was synthesized (Figure S1A). The detailed NMR data are described in the Supplementary files (Figure S1B). The synthesis of azide-modified MMs and CGA-modified MMs is shown in Figure S2. The confirmation of CGA modification on the surfaces of MMs was demonstrated by LC–MS (Liquid Chromatography-Mass Spectrometry), and is shown in Table S1 and Figure S3. The CGA-modified MMs were added to the cell lysates and incubated for 10 h at 4 °C. The MMs were collected by magnetic separation, and rinsed three times with precooled PBS. Then, the captured proteins were released by incubation with 200 µL of DL-dithiothreitol (DTT; 100 μM) at 4 °C for 30 min. Finally, the supernatants were collected for sodium dodecyl sulfate-polyacrylamide gel electrophoresis (SDS-PAGE) or Western blot analysis.

### 2.7. Co-Localization of CGA with Target Protein

HepG2 cells were randomly divided into four groups: control, CGA, alkynyl-CGA, and CGA + alkynyl-CGA. Cells were cultured to approximately 70% confluence, and then treated with or without CGA and alkynyl-CGA for 6 h, as indicated. In the CGA and alkynyl-CGA groups, cells were treated with 10 μM CGA or alkynyl-CGA. In the CGA + alkynyl-CGA group, cells were treated with 100 μM CGA and 10 μM alkynyl-CGA. Then, immunofluorescence cytochemistry was performed. Briefly, the treated cells were fixed in 4% paraformaldehyde for 15 min, and then washed with PBS. After blocking with 10% goat serum for 1 h, the cells were incubated with anti-AKT antibody (1:1000) at 4 °C overnight. Cells were then labeled with Alexa Fluor^®^ 594-conjugated goat anti-rabbit IgG (1:500) for 1 h at 37 °C and observed under a confocal microscope. The excitation and emission wavelengths of Alexa Fluor^®^ 594 were 590 nm and 617 nm, respectively. After washing, a click chemistry reaction of N_3_-tag with alkynyl-CGA was carried out in these fixed cells to obtain a CGA-fluorescent probe, in accordance with our previously established method (details in Supplementary Materials and Figure S4). The excitation and emission wavelengths of the CGA-fluorescent probe were 365 nm and 480 nm, respectively.

### 2.8. Competitive Test against AKT Using CGA-Modified MMs

To elucidate the interaction mechanism between CGA and AKT, we used a competitive test to evaluate the combination mode. Different AKT ligands, such as SC79, PHT-427, AT7867, and AKT inhibitor VIII, were respectively chosen to compete with the CGA probe against CGA-modified MM-captured AKT protein. Briefly, HepG2 cell pellets were homogenized in RIPA lysis buffer. Fifty microliters of the lysate was added to 1 mL of azide-modified MMs, or 250 μL was added to 5 mL of CGA-modified MMs. The mixtures were incubated with gentle shaking at 4 °C overnight. Then, the proteins bound with MMs were collected by magnetic separation, washed three times with precooled PBS, and resuspended. The lysate only, incubated with azide-modified MMs, served as the blank group. In the DTT-positive group, the captured AKT proteins were released with 250 μL of DTT (100 μM). In the remaining four groups, 200 μL of the same dose of competitors (100 μM) was added to compete with the CGA-modified MM probe. Finally, 10 μL of the supernatants containing dissociated AKT was collected from each group, and the AKT concentration was determined using SDS-PAGE and Western blot.

### 2.9. Membrane Translocation of the PH-Domain

The pcDNA3-AKT-PH-GFP plasmid (PH-GFP) and pcDNA3-AKT-PH(R25C)-GFP plasmid (R25C) were used to show the localization of the AKT PH domain on the membrane. Six groups of HEK 293T cells were cultured to approximately 50% confluence. Then, they were treated with SC79 or CGA (10, 1, and 0.1 μM) for 6 h. After washing with PBS, cells were transfected with PH-GFP for 22 h, except for the cells in the R25C group, which were transfected with R25C plasmid instead. Images of GFP fluorescence were captured with a spectral-type AF7000 Live Cell Imaging System (Carl Zeiss, Oberkochen, Germany). The excitation and emission wavelengths of GFP were set as 488 nm and 507 nm, respectively. The relative fluorescence of AKT-PH-GFP on the HEK 293T membrane from eight cells per group was determined.

### 2.10. SDS-PAGE and Western Blot Analysis

Briefly, treated cells were washed twice with ice-cold PBS and lysed with lysis buffer for 0.5 h on ice. Cell lysates were centrifuged at 10,000× *g* for 15 min at 4 °C to obtain the supernatants. Then, the supernatants were mixed with loading buffer, boiled, and loaded onto SDS-PAGE, and subject to Coomassie Blue staining where appropriate. SDS-PAGE gels were transferred to polyvinylidene fluoride (PVDF) membranes for Western blot analysis. The membranes were blocked with 1% skim milk at room temperature for 1 h, and then incubated with primary antibodies at 4 °C overnight. Membranes were then incubated with their respective secondary antibodies at room temperature for 1 h. Finally, the membranes were incubated with chemiluminescent HRP substrates and exposed in a Tanon-5200 Chemiluminescence Apparatus (Shanghai, China). The relative optical band densities were quantified using the software ImageJ.

### 2.11. Statistical Analysis

Data were presented as the mean ± standard error of the mean (SEM) for the number of observations. One-way analysis of variance (ANOVA), followed by the a Dunnett post hoc test using the SPSS 11.5 program (SPSS Inc., Chicago, IL, USA), was used for comparison among the groups. Differences were considered statistically significant when *p*-values were <0.05.

## 3. Results

### 3.1. CGA Regulates Glucose Metabolism by Increasing AKT Activity

**Figure 1:**
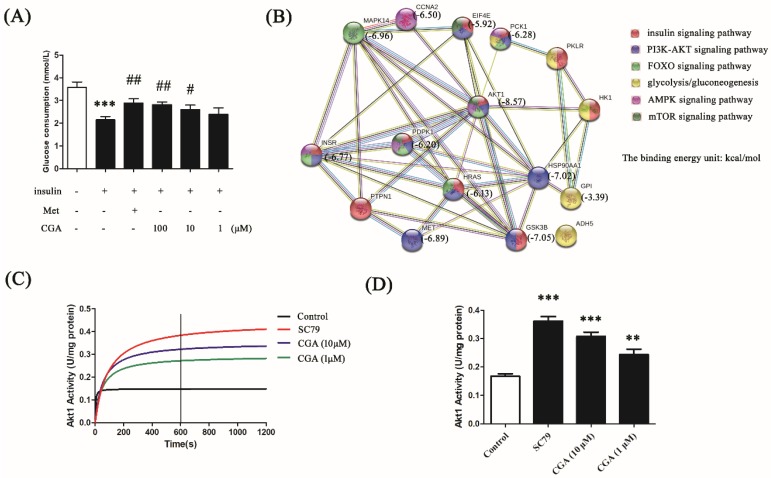
The effects of chlorogenic acid (CGA) on the regulation of glucose consumption and the prediction of its target proteins. (**A**) Effects of different concentrations of CGA on glucose consumption in insulin-treated HepG2 cells. Metformin hydrochloride (Met) served as a positive control. Each bar represents the mean ± standard error of the mean (SEM). +: with; -: without. *** *p* < 0.001 vs. control; # *p* < 0.05 vs. model; ## *p* < 0.01 vs. model (*n* = 6). (**B**) Prediction of the targets of CGA using PharmMapper and analysis of the interaction and signaling on potential target proteins using String v9.1. (**C**) The enzymatic activity of protein kinase B (AKT) in HepG2 cells after treatment with different concentrations of CGA. SC79 was used as a positive control. (**D**) The histogram presents the activities of AKT at 600 s after the respective treatments. Each bar represents the mean ± SEM. ** *p* < 0.01 vs. control; *** *p* < 0.001 vs. control (*n* = 3).

In our study, glucose consumption was used as an index to evaluate the effect of CGA on glucose metabolism in the in vitro model. Insulin treatment induced a significant decrease in glucose consumption in HepG2 cells (Figure 1A). CGA reversed this in a dose-dependent manner, confirming its role in regulating glucose metabolism.

Using the PharmMapper software, the potential target proteins of CGA were obtained based on its three-dimensional structure. A string analysis was performed among the 16 targets which participate in the glucose and lipid metabolism pathways, i.e., the insulin signaling pathway, the PI3K/AKT signaling pathway, the FOXO signaling pathway, glycolysis/gluconeogenesis, the AMP-activated protein kinase (AMPK) signaling pathway, and the mammalian target of rapamycin (mTOR) signaling pathway (Figure 1B). Among these predicted target proteins, AKT was shown to participate in five of the six pathways mentioned above, and presented the possibility of binding, based on the predicted energy of binding (−8.57 kcal/mol). Therefore, AKT was chosen as the predicted target protein of CGA for further study. The regulation of AKT by CGA was also confirmed by AKT enzymatic activity assay. As shown in Figure 1C,D, CGA significantly increased AKT activity, suggesting the possibility of AKT as a target of CGA in regulating glucose metabolism.

### 3.2. Identification of AKT as a Target of CGA

**Figure 2:**
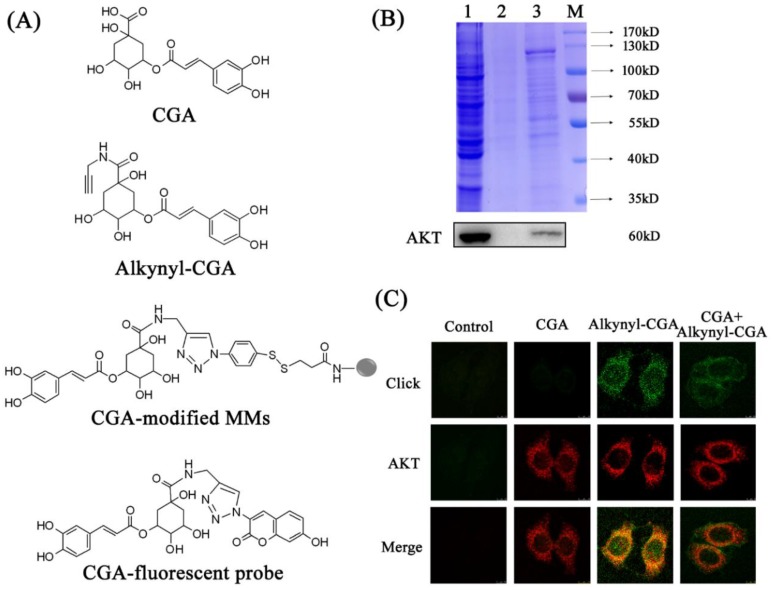
The chemical structures of the compounds used and results of target fishing. (**A**) The chemical structures of CGA, alkynyl-CGA, CGA-modified magnetic microspheres (MMs), and the CGA-fluorescent probe. (**B**) SDS-PAGE (upper panel) and Western blot analysis (lower panel) were used to detect proteins enriched by CGA-modified MMs in HepG2 cells. Lane 1: total protein content of the HepG2 cell lysate. Lane 2: the protein enriched from HepG2 cell lysate by azide-modified MMs. Lane 3: the protein enriched from HepG2 cell lysate by CGA-modified MMs. M: marker. (**C**) Co-localization of the CGA-fluorescent probe (green) and AKT antibody (red) in HepG2 cells (magnification 200×).

To make target fishing easier, an alkynyl-modified CGA probe (alkynyl-CGA) tracer was used. Alkynyl modification showed no significant alteration to CGA activity, as measured by glucose consumption (Figure S1C). The chemical structures of CGA, alkynyl-CGA, CGA-modified functionalized MMs, and the CGA-fluorescent probe are presented in Figure 2A.

CGA-modified MMs were used in target fishing, and SDS-PAGE and Coomassie Brilliant Blue staining were performed to evaluate the collection efficiency. The total amount of protein in HepG2 cell lysate was detected, and is shown as Lane 1 in the upper panel of Figure 2B. CGA-modified MMs were shown to enrich some proteins from the cell lysate (Lane 3), in contrast to the negative control bands in Lane 2 (only azide-modified MMs), which were much weaker. This result indicates that these enriched proteins were selectively captured due to CGA. Then, the proteins from the same SDS-PAGE gel were transferred to PVDF membranes for Western blot analysis, and a clear band of AKT was detected (lower panel, Figure 2B). The result verified the specificity of the binding between AKT and CGA.

Next, co-localization of AKT and CGA was performed to demonstrate the possibility of binding between them. The CGA-fluorescent probe, the product obtained in the click reaction of alkynyl-CGA and N_3_-tag, was used to display the distribution of CGA in HepG2 cells. As shown in Figure 2C, the conjugate ring in the CGA-fluorescent probe showed strong green fluorescence, and the red fluorescence of Alexa Fluor^®^ 594 reflected the distribution of AKT. Neither alkynyl-CGA nor the N_3_-tag itself showed any fluorescence before the click reaction (Figure S4B). More importantly, in the CGA + alkynyl-CGA group, the intensity of the green fluorescence was reduced, due to competition from the native CGA compound. In the control group, the click reaction and immunofluorescence cytochemistry were performed as in the other groups, except that no CGA, alkynyl-CGA, or primary antibody for AKT were added. As a result, no green or red fluorescence was observed. This excluded the existence of any possible artefactual background fluorescence, and assured the specificity of the results. These results demonstrate that the hypothesis that CGA targets AKT, and has the same location as AKT in HepG2 cells is reasonable.

### 3.3. CGA Binds to the PH Domain of AKT

**Figure 3:**
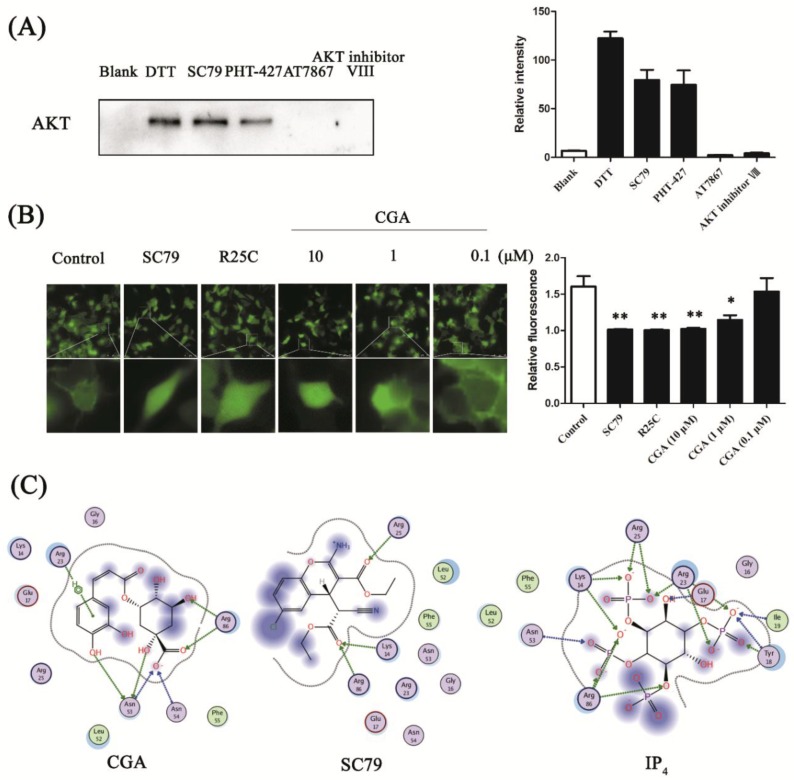
Confirmation of CGA binding to the AKT pleckstrin homology (PH) domain and the predicted interaction pose. (**A**) Competition tests of SC79, PHT-427, AT7867, and AKT inhibitor VIII with the CGA probe against enriched AKT by CGA-modified functionalized MMs. Bands of AKT were detected by Western blot. Blank: fished proteins by azide-modified MMs; DL-dithiothreitol (DTT): released proteins by DTT from CGA-modified functionalized MMs. SC79, PHT-427, AT7867, and AKT inhibitor VIII samples were replaced from CGA-modified functionalized MMs by treatment with SC79, PHT-427, AT7867, and AKT inhibitor VIII, respectively. The histogram presents the relative intensities of the AKT bands (*n* = 3). (**B**) Representative images showing the membrane translocation of AKT PH with and without CGA treatment (upper row, 40×; lower row, 200×). The histogram presents the relative fluorescence of AKT-PH-GFP on the HEK 293T membrane. Each bar represents the mean ± SEM. * *p* < 0.05 vs. control; ** *p* < 0.01 vs. control (*n* = 8 cells). (**C**) Two-dimensional interaction map for CGA, SC79, and inositol (1,3,4,5)-tetrakisphosphate (IP_4_) docked with AKT PH-domain-binding sites, respectively (Protein Data Bank (PDB): 1UNQ).

To clarify the details of the binding pocket of AKT with CGA, a set of AKT ligands was chosen to compete with CGA-modified MMs in capturing AKT. SC79 and PHT-427 are reported to be a selective stimulator and an inhibitor of AKT, respectively, which combine with the AKT PH domain [26,27]. AT7867 is an inhibitor of AKT which binds at the ATP binding pocket of AKT [28]. AKT inhibitor VIII is an allosteric inhibitor of AKT [29]. As shown in Figure 3A, compared with the DTT group, the bands of AKT in the SC79 and PHT-427 groups were clearly detected by Western blot, while no band was seen in the AT7867 or AKT inhibitor VIII groups. This result indicates that CGA might combine with the AKT PH domain in a similar manner to SC79 and PHT-427.

To further confirm the hypothesis, we tested the membrane translocation of the AKT PH domain with and without CGA treatment. In the HEK 293T cells of the control group transfected with PH-GFP, green fluorescence was observed on the membrane, showing that a part of the overexpressed AKT PH was transferred to the plasma membrane. The results showed that the translocation was inhibited by the AKT activator SC79, which can suppress AKT plasma membrane translocation with enhanced AKT phosphorylation and activation [26]. Meanwhile, R25C, the AKT PH-domain mutant carrying an arginine to cysteine mutation at amino acid 25, abrogated the association with the plasma membrane as it does not bind to phosphatidylinositol (3,4,5)-trisphosphate (PIP_3_) [30]. SC79 and R25C were used as positive controls to show the inhibition of membrane translocation (Figure 3B). The membrane translocation of AKT PH was also shown to be hampered by CGA in a dose-dependent manner, confirming that CGA might bind to the PH domain of AKT, similarly to SC79 (Figure 3B).

To provide additional insights into the interaction of CGA and the AKT PH domain, we docked CGA, SC79, and IP_4_ as ligands into the binding site of the AKT PH domain (PDB: 1UNQ) using MOE software. After virtual simulation, the ligands were docked with the target protein to determine the best docking model with the lowest energy level. We then analyzed the top scoring poses of CGA in all cases, which are displayed as two-dimensional (2D) maps, indicating the interactions of CGA (−6.8855 kcal/mol), IP_4_ (−5.5429 kcal/mol), or SC79 (−5.2175 kcal/mol) with the AKT PH domain (Figure 3C).

Lys-14, Arg-25, and Arg-86 of AKT PH are regarded as the key amino residues closely related to membrane translocation, through hydrogen-bond interactions [31]. As shown in Figure 3C, hydrogen bonds were predicted between the residues of Asn-53, Asn-54, Arg-86, and CGA, and the activator SC79 was associated with the Lys-14, Arg-25, and Arg-86 side-chain residues through hydrogen bonds. In addition, hydrogen bonds formed between IP_4_, the natural ligand of AKT, and the Lys-14, Tyr-18, Arg-23, Arg-25, and Arg-86 side-chain residues. The molecular docking result explained the mechanism of AKT activation by CGA, which might be presenting a suitable interaction pose in the AKT PH domain, as compared with SC79 and IP_4_.

### 3.4. CGA Phosphorylates AKT and Regulates Its Downstream Factors

**Figure 4:**
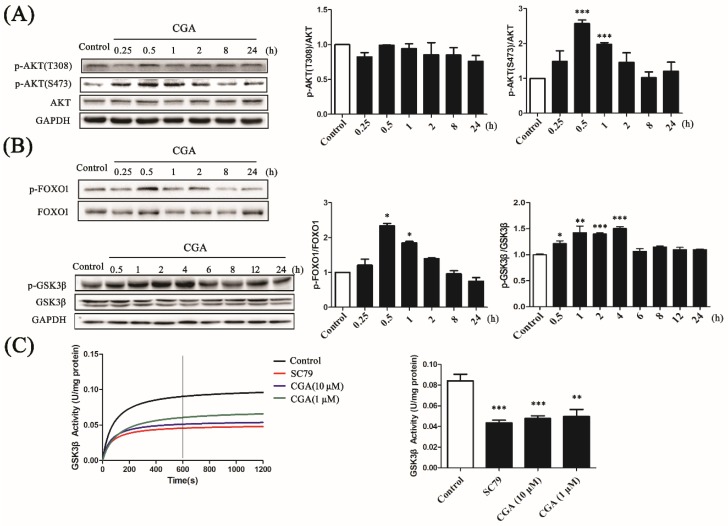
The effects of CGA on AKT phosphorylation and its downstream factors. (**A**) The time-dependent effect of CGA on the phosphorylation of AKT (T308 and S473). (**B**) The time-dependent effect of CGA on the phosphorylation of forkhead box O1 (FOXO1) and glycogen synthase kinase 3β (GSK3β). The histograms present the relative intensities of the detected protein bands. (**C**) The effect of CGA on the enzymatic activity of GSK3β. SC79 was used as the positive control. The histogram presents the activity of GSK3β at 600 s after the respective treatments. Each bar represents the mean ± SEM. * *p* < 0.05 vs. control; ** *p* < 0.01 vs. control; *** *p* < 0.001 vs. control (*n* = 3).

To evaluate the effect of CGA on the downstream molecules, we detected AKT phosphorylation at Thr-308 (T308) and Ser-473 (S473). Interestingly, the ratio of p-AKT (S473)/AKT was markedly increased by CGA in the interval between 0.5 h to 1 h; however, the ratio of p-AKT (T308)/AKT was not (Figure 4A), indicating specific regulation of p-AKT (S473) by CGA. Being downstream factors of AKT, the phosphorylation of FOXO1 and GSK3β also changed in accordance with p-AKT (S473). As shown in Figure 4B, CGA induced phosphorylation of FOXO1 and GSK3β in a time-dependent manner. In addition, the enzymatic activity of GSK3β was analyzed to further confirm the effect of CGA on AKT’s downstream mediators. CGA significantly downregulated the activity of GSK3β (Figure 4C), coinciding with the phosphorylation of GSK3β. These results demonstrated that CGA could partly phosphorylate AKT and regulate its downstream factors.

## 4. Discussion

In previous work, CGA was demonstrated to show beneficial effects on the regulation of glucose and lipid metabolism disorders [7,8,11,12]. Han et al. reported that CGA could protect mouse osteoblastic MC3T3-E1 cells from oxidative damage via activating the PI3K/AKT-mediated pathway [32]. Ong et al. reported the activation of AKT by CGA during the stimulation of glucose transport in skeletal muscle [7]. However, how CGA interacts with AKT in the regulation of glucose and lipid metabolism remains unclear. In our study, we focused on the regulation of glucose metabolism by CGA.

It is known that AKT possesses a PH domain at the N-terminus, a kinase domain in the middle, and a regulatory domain at the C-terminus. The activation of AKT commonly relies on membrane translocation and subsequent phosphorylation by the kinase recruited to the membrane. For example, PI(3,4,5)P_3_, a second messenger, binds to the AKT PH domain and causes plasma membrane targeting, leading to the phosphoactivation of AKT by pyruvate dehydrogenase kinase 1 (PDK1) [33]. Interestingly, membrane translocation is not always needed for AKT activation. For example, SC79 prohibits AKT from translocating to the membrane, but is an AKT activator [26]. Our enzymatic activity analysis of AKT confirmed the activation of AKT by CGA. In our competition test, CGA was shown to activate the AKT PH domain with the same pattern as SC79.

The interactions between small molecules and their target proteins are mainly due to hydrogen bonds, hydrophobic forces, and changes in the hydrophobicity of the surrounding microenvironment due to small molecules [34]. In a previous study regarding the structure of the PH domain of AKT bound to PI(3,4,5)P_3_, three amino acid residues, Lys-14, Arg-25, and Arg-86, were reported to be critical for AKT activation [31]. For example, the mutation of Arg-86 (which interacts with the D4 phosphate group) to Ala completely abolished PI(3,4,5)P_3_ and PI(3,4)P_2_ binding to AKT. Our result showed that the molecular docking of IP_4_ with the AKT PH domain is in good accordance with previous reports, and SC79 was shown to bind to the AKT PH domain at all three residues, in accordance with its AKT activation effect (Figure 3C). However, CGA was predicted to bind to the AKT PH domain at the critical residue Arg-86, as IP_4_ and SC79 did. In addition, other amino acid residues, such as Arg-23 and Asn-53, were also reported to be important, as the interaction of Arg-23 with the D1 phosphate plays a role in regulating the overall affinity of AKT for 3-phosphoinositides. In addition, a slight reduction in binding was observed for the Asn-53 mutation (which interacts with the D3 and D4 phosphates) [31]. Molecular docking provided insight into the interaction between CGA and the AKT PH domain, displayed a possible embedding mode in the active pockets, and explained the mechanism of AKT activation by CGA.

Although the membrane translocation of AKT is hampered, the AKT structure can still be transformed after SC79 binding to the PH domain; thus, the phosphorylation sites are exposed and ready for activation [26]. Since CGA shares a similar mechanism to AKT activation, we next examined AKT phosphorylation by CGA. With the binding of CGA to the PH domain, AKT was shown to be phosphorylated at S473, but not T308, and activated. This can be explained by the S473 and T308 phosphorylation sites being targeted by different kinases, namely PDK1 and mTORC2 (mammalian target of rapamycin complex 2), respectively [35]. Although the maximum activation of AKT is supposed to rely on the phosphorylation at both S473 and T308, only the phosphorylation at S473 is reported to stimulate AKT activation. For example, Case et al. reported that the phosphorylation of AKT at S473, caused by mechanical input, was sufficient to cause the inactivation of GSK3β in mesenchymal stem cells [36]. As shown in our results, the phosphorylation of both GSK3β and FOXO1 was significantly upregulated by CGA treatment, accompanied by downregulation of the enzymatic activity of GSK3β. These results indicate that CGA might regulate glucose and lipid metabolism by directly combining with AKT to regulate its downstream mediators.

In conclusion, our findings demonstrate that CGA could regulate glucose metabolism by directly targeting AKT. After CGA binds to the PH domain of AKT at its critical activation site, it is phosphorylated at S473 and activated, despite AKT being hampered from binding to the cell membrane. The phosphorylation of the downstream proteins, namely GSK3β and FOXO1, facilitates the regulation of glucose metabolism by CGA. As CGA exists in the human diet, for example, in drinks, fruits, and vegetables, our results provide some evidence for the application of nutrition containing CGA as a complementary therapy for glucose metabolism disorders.

## Supplementary Materials

The following are available online at http://www.mdpi.com/2072-6643/10/10/1366/s1: Figure S1: Synthesis of alkynyl-modified CGA probe; Figure S2: The synthetic route for functionalized azide-MMs and CGA-modified MMs; Figure S3: First-step mass spectrum of the solution of CGA-modified functionalized MMs after DTT reduction; Figure S4: Synthesis of CGA-fluorescent probe; Table S1: Mass spectral data of three compounds.
