# Supplementary material for: Chlorogenic Acid Targeting of the AKT PH Domain Activates AKT/GSK3β/FOXO1 Signaling and Improves Glucose Metabolism
Source: Nutrients. 2018 Sep 23;10(10):1366. doi: 10.3390/nu10101366 (PMC6212807; doi:10.3390/nu10101366)
Supplement: Supplementary file 1 [file nutrients-10-01366-s001.pdf]

Supplementary Materials: Chlorogenic Acid Targeting of the AKT PH Domain Activates AKT/GSK3β/FOXO1 Signaling and Improves Glucose Metabolism

Jie Gao [1], Xin He [1], Yuejiao Ma [1], Xuezhi Zhao [1], Xiaotao Hou [2], Erwei Hao [2], Jiagang Deng [2] and Gang Bai [1,*]

1. Synthesis of Alkynyl-Modified CGA Probe

Chlorogenic acid (1 g, 2.8 mmol) was added to a stirred solution of 2-propynylamine (155.4 mg, 2.8 mmol) and hexafluorophosphate azabenzotriazole tetramethyl uronium (HATU; 1 g, 2.8 mmol) in 40 ml of dimethylformamide (DMF). The mixture was stirred under argon at room temperature for 16 h. The reaction product was detected by thin-layer chromatography (TLC; dichloromethane:methanol = 3:1, Rf = 0.6). Subsequently, the mixture was diluted with water (75 ml) and extracted with ethyl acetate. Next, anhydrous sodium sulfate was appended to the collected organic phase for desiccation, filtration, and concentration under vacuum. Finally, the mixture was concentrated in vacuo and purified by column chromatography on silica gel (dichloromethane:methanol = 30:1) to obtain the alkynyl-modified CGA probe (Figure S1A). It is a faint-yellow solid. The yield of this reaction was 6.3% (180 mg, 0.24mmol). 1H nuclear magnetic resonance (1H NMR) spectra were recorded on a Bruker AV 400 spectrometer at 400 MHz (Figure S1B), using dimethyl sulfoxide (DMSO) as solvent. Coupling constants are reported in Hertz. The detailed NMR data are as follows: ET11731-1-P1A 400 MHz DMSO-d6 δ = 8.16 (t, J = 6.0 Hz, 1H), 7.47 (d, J = 16.0 Hz, 1H), 7.03 (s, 1H), 6.98–7.00 (m, 1H), 6.76 (d, J = 8.0 Hz, 1H), 6.23 (d, J = 16.0 Hz, 1H), 5.59 (br s, 1H), 5.19–5.26 (m, 1H), 4.07 (d, J = 2.4 Hz, 1H), 3.80–3.82 (m, 2H), 3.55–3.58 (m, 1H), 3.01–3.02 (m, 1H), 1.73–1.98 (m, 4H).

Glucose consumption assay of HepG2 cells was used to determine the activity of CGA after alkynyl modification. After insulin induction, HepG2 cells in different treatment groups were treated with CGA or alkynyl-modified CGA (10−5, 10−6, and 10−7mol/L) for another 12 h. Met (10−5mol/L) was used as a positive control. Then, the supernatants were collected for glucose concentration assay as described in Section 2.3 in the manuscript. As shown in Figure S1C, insulin treatment induced a significant decrease in glucose consumption in HepG2 cells. CGA (10−5 and 10−6 mol/L) could reverse it, the same as Met did. Alkynyl-modified CGA at the same concentration as CGA exerted the similar effect in regulating glucose consumption. This indicated that the alkynyl modification did not change CGA activity. Thus, alkynyl-modified CGA could be used as a substitute for CGA in the following experiments.

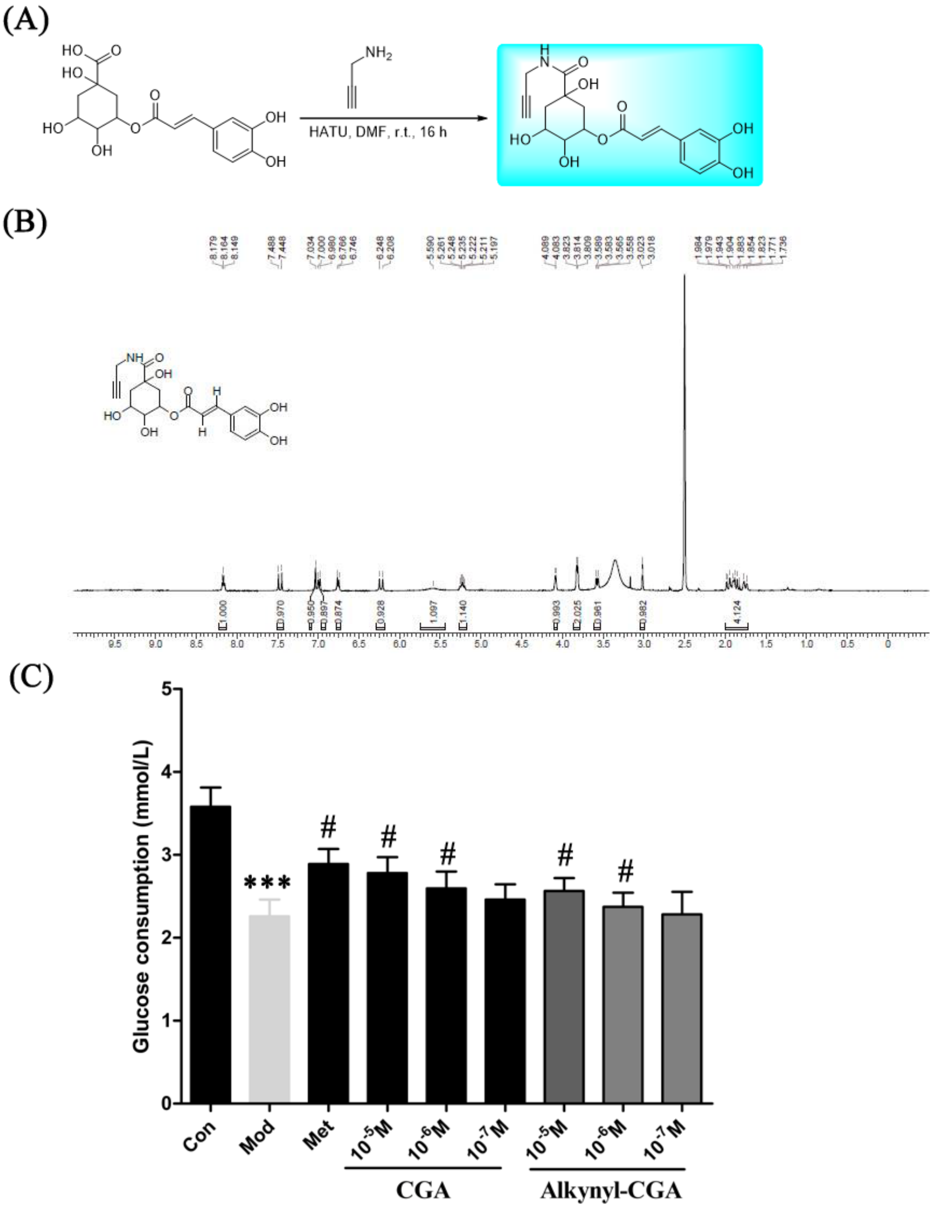

Figure S1. Synthesis of alkynyl-modified CGA probe. (A) Synthetic route for alkynyl-modified CGA probe. (B) The ^{1}H NMR data of alkynyl-modified CGA probe (400 MHz, dimethyl sulfoxide (DMSO)). (C) Alkynyl modification did not influence the pharmacological effect of CGA in glucose consumption. Met served as a positive control. Each bar represents the mean ± SEM. *** $p < 0.001$ vs. control; $^{\#}$ $p < 0.05$ vs model (n = 6).

2. Preparation of CGA-Modified Functionalized MMs

Firstly, 0.36 mL of 10 mg/mL Sulfo-SADP (sodium1-((3-((4-azidophenyl) disulfanyl) propanoyl) oxy)-2,5- dioxopyrrolidine-3- sulfonate; Bioworld, MN, USA) and 18 mL of 5 mg/mL Fe_3O_4 amino magnetic microspheres (NH_2-MMs; Tianjin Baseline Chromtech Research Center, Tianjin, China) were added in 5 mL of borate buffer and reacted for 18 h at room temperature to obtain the azide-modified MMs. The azide-MMs were enriched via magnetic separation, washed three times with redistilled water, and then three times with methanol. Three milliliters of functionalized azide-MMs were collected as the control group and the others were used directly for the next steps. Alkynyl-modified CGA (10^{-4} mol/L) was added to the functionalized azide-MMs and incubated with catalyst solution (10^{-3} mol/L $CuSO_4$ and 10^{-3} mol/L sodium ascorbate in pre-cooled PBS). The reaction mixture was shaken at room temperature in the dark for 24 h. After fully interacting, the CGA-modified functionalized MMs were separated with a magnet and washed with methanol and redistilled water.

The synthetic route for functionalized azide-MMs and CGA-modified MMs is shown in Figure S2.

The enriched CGA-modified MMs were reduced by DL-dithiothreitol (DTT) (10^{-4} mol/L) (Figure S2). The released CGA derivative was analyzed by a liquid chromatograph/mass spectrometer (LC–MS, Shimadzu 2020, Japan). The results are shown in Table S1 and Figure S3. LC–MS (electrospray ionization (ESI)): *m*/*z* [M − H] calculated for $C_{25}H_{26}N_4O_8S$: 542; found: 541. The results of LC–MS demonstrated that CGA was successfully modified on the surfaces of MMs.

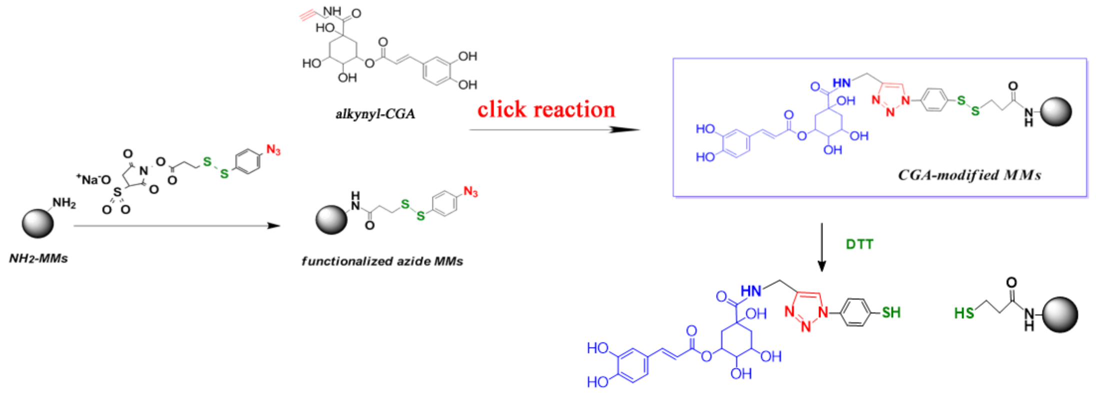

Figure S2. The synthetic route for functionalized azide-MMs and CGA-modified MMs. The obtained compounds from CGA-modified MMs after DTT reduction are also shown.

Table S1. Mass spectral data of the three compounds. MW—molecular weight; CGA—chlorogenic acid; DTT— DL-dithiothreitol; ESI—electrospray ionization.

Compound	Relative MW	Chemical formula	[M – H]⁻ (*m*/*z*)	ESI (+/−)
CGA	354.31	$C_{16}H_{18}O_9$	353.21	ESI (−)
Alkynyl-CGA	391.37	$C_{19}H_{21}NO_8$	390.11	ESI (−)
Product after DTT reduction	542.56	$C_{25}H_{26}N_4O_8S$	541.13	ESI (−)

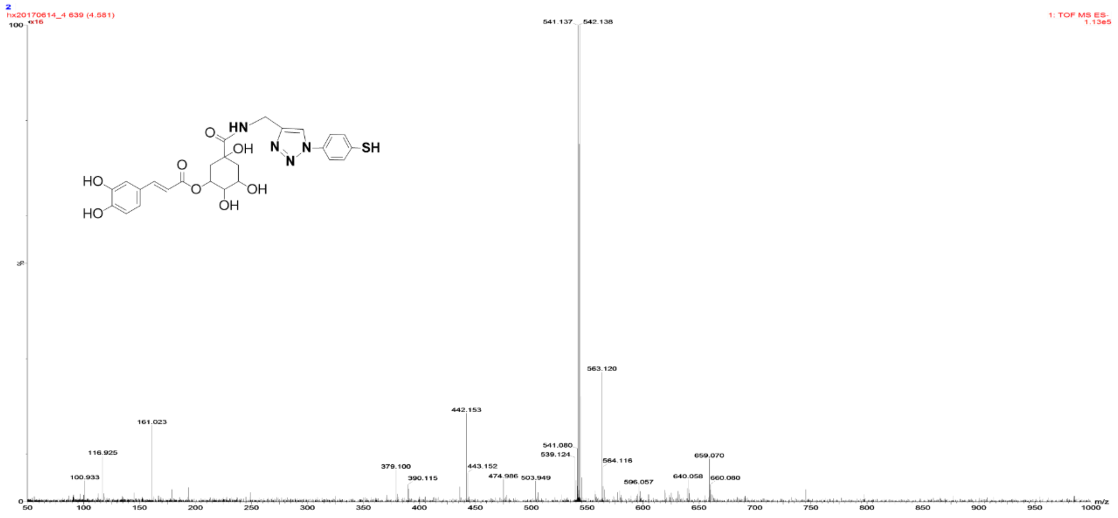

Figure S3. First-step mass spectrum of the solution of CGA-modified functionalized MMs after DTT reduction.

3. Click Chemistry Reaction of CGA Fluorescent Probe

A click chemistry reaction of alkynyl-modified CGA and N3-tag in cells was performed to obtain the CGA fluorescent probe. Briefly, HepG2 cells were cultured in a 20-mm culture dish with 10–5 mmol/L alkynyl-modified CGA probe for 6 h. The supernatant was removed, and cells were washed with precooled PBS twice for 15 min each time. Then, cells were fixed using 4% paraformaldehyde

for 15 min, and later incubated with a 100-µL reaction solution (0.2 mmol/L N3-tag, 0.2 mmol/L tristriazoleamine, 1.0 mmol/L CuSO4, and 2.0 mmol/L sodium ascorbate in precooled PBS). The reaction is shown in Figure S4A. After 1 h of incubation, the cells were washed with PBS-Tween (PBST). The maximum absorption of the product was shown to be at 480–485 nm by full wavelength scanning. While alkynyl-CGA showed no fluorescence before the click reaction, neither did N3-tag itself (Figure S4B). The fluorescence images were taken under a confocal microscope (Carl Zeiss, Oberkochen, Germany). The excitation and emission wavelengths chosen were 365 nm and 480 nm, respectively. The representative image of the fluorescence in cells is shown in Figure S4B.

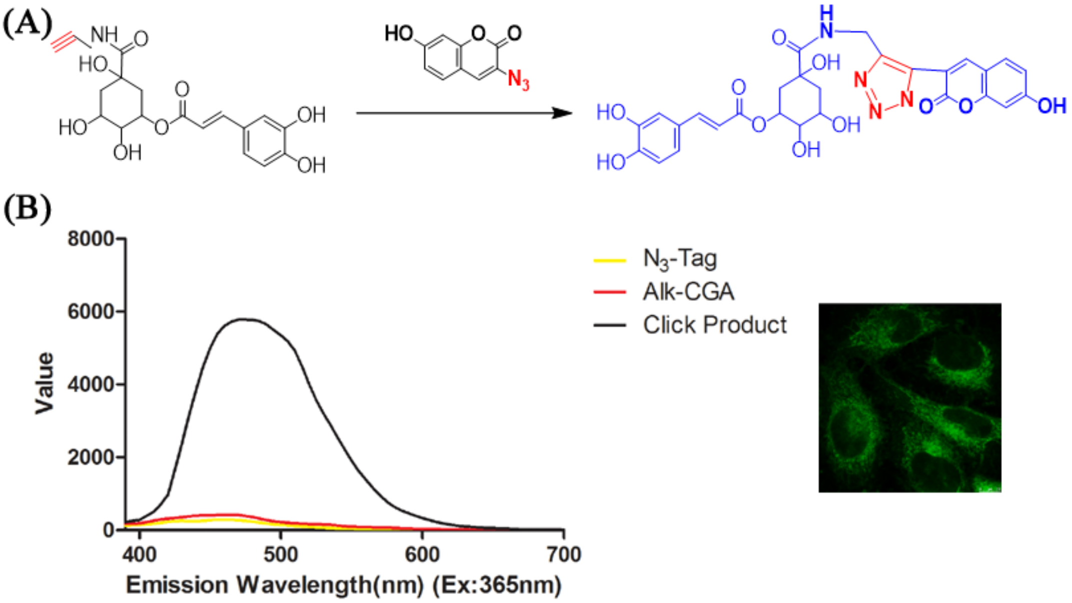

Figure S4. Synthesis of CGA-fluorescent probe. (A) The synthetic reaction of alkynyl-modified CGA and N3-tag to obtain the CGA-fluorescent probe (click product). (B) Left panel: the fluorescence intensity of alkynyl-modified CGA, N3-tag, and click product by full wavelength scanning. Right panel: a representative image showing the CGA-fluorescent probe in HepG2 cells after the click reac.
